# Supportive Accountability: A Model for Providing Human Support to Enhance Adherence to eHealth Interventions

**DOI:** 10.2196/jmir.1602

**Published:** 2011-03-10

**Authors:** David C Mohr, Pim Cuijpers, Kenneth Lehman

**Affiliations:** ^2^Department of PsychologyVrije UniversiteitAmsterdamNetherlands; ^1^Department of Preventive MedicineNorthwestern UniversityChicago, ILUnited States

**Keywords:** Internet intervention, adherence, computer-mediated communication, accountability, human support, motivation

## Abstract

The effectiveness of and adherence to eHealth interventions is enhanced by human support. However, human support has largely not been manualized and has usually not been guided by clear models. The objective of this paper is to develop a clear theoretical model, based on relevant empirical literature, that can guide research into human support components of eHealth interventions. A review of the literature revealed little relevant information from clinical sciences. Applicable literature was drawn primarily from organizational psychology, motivation theory, and computer-mediated communication (CMC) research. We have developed a model, referred to as “Supportive Accountability.” We argue that human support increases adherence through accountability to a coach who is seen as trustworthy, benevolent, and having expertise. Accountability should involve clear, process-oriented expectations that the patient is involved in determining. Reciprocity in the relationship, through which the patient derives clear benefits, should be explicit. The effect of accountability may be moderated by patient motivation. The more intrinsically motivated patients are, the less support they likely require. The process of support is also mediated by the communications medium (eg, telephone, instant messaging, email). Different communications media each have their own potential benefits and disadvantages. We discuss the specific components of accountability, motivation, and CMC medium in detail. The proposed model is a first step toward understanding how human support enhances adherence to eHealth interventions. Each component of the proposed model is a testable hypothesis. As we develop viable human support models, these should be manualized to facilitate dissemination.

## Introduction

It is widely recognized that eHealth interventions are often plagued by a high rate of attrition [1-3]. While a wide variety of factors such as the design of the eHealth intervention and patient factors have been suggested as potential factors in adherence and attrition [1, 4], support provided by clinicians or coaches, via telephone, email, and chat rooms, has been shown across many treatment targets to enhance adherence [3,5-7].

However, very little attention has been paid to *how* human interaction enhances adherence. The aim of this paper is to propose a theoretical model, which we call “Supportive Accountability,” that can serve as the basis for a “science of adherence” [1] for human support. A clear theoretical model would provide intervention developers and researchers with a starting point for future research, as well as the basis for a more structured and manualized approach to design and implementation of human support intervention components.

A few basic definitions must be established, as terminologies may take on subtly different meanings from how they are used in traditional, face-to-face interventions. *Adherence* is defined here as use of the eHealth intervention over time, and has been operationalized in a variety of ways such as number of logins, time on site, number of modules completed, and number of characters typed into the site [3,8]. This definition emphasizes how users of eHealth interventions are assumed to be active patients insofar as they log in to or otherwise access the resource as a period of behavior change is progressing. It should be noted that this definition focuses on adherence to the eHealth intervention, and not adherence to any behavioral prescription. While adherence to behavioral prescriptions is critical to the success of psychological interventions [9], it is beyond the scope of this paper.

*Support*, in our nomenclature, may be provided by a range of people, including lay persons, students, mental health professionals, and medical professionals. We will use the term *coach* to refer to the support person, as it carries no implications regarding background. Indeed, specific lay coaches may be just as effective as professionals in supporting eHealth interventions [10].

## Current Models From Face-to-Face Psychological and Behavioral Treatments

Adherence has been called the paramount issue in psychological treatments [11]. More than 50% of patients receiving psychological interventions in clinical settings have been found to drop out of treatment prematurely [12,13]. Even in structured randomized controlled trials (RCTs) with rigorous patient selection and extra support of research staff, 15%-30% attrition is common [14,15]. Despite such rates, there is little literature on the causes of attrition, and even less on how to prevent it. What research does exist suggests that patients terminate prematurely primarily due to poor therapeutic alliance [16] and patient variables, such as diagnosis of a personality disorder [11,17]. Surprisingly, to the best of our knowledge, there is no overarching theoretical framework for examining adherence. Part of the reason for this may be that for standard face-to-face behavioral treatments, procedures aimed at enhancing adherence are embedded in the treatment itself. This is to say, in face-to-face interventions, the treatment provider offers the core of the intervention while simultaneously coordinating his or her relationship with the patient in a way that will efficiently promote the use of the therapeutic skills and interest to continue in treatment. In contrast, eHealth treatments separate the content of the treatment, which is provided in a standardized manner via a website, mobile device, or other platform, from support provided by humans, which is often intended to increase adherence [18-20].

Constructs generally examined in association with adherence in the face-to-face treatment literature do not adequately explain why such support might improve adherence. For example, emotional bond or therapeutic alliance is nearly universally acknowledged as important for adherence in almost any form of psychological, behavioral, or medical procedure. But these constructs do not elucidate the mechanisms by which bond or therapeutic alliance might lead to increased adherence. Clearly, many treatments, such as motivational interviewing [21], aim to promote adherence. Yet our review of the clinical literature found a dearth of useful theory to apply to the problem of adherence in eHealth interventions.

A broader review of related literature, however, revealed much useful information. Organizational psychology has long examined how to obtain adherence to behavioral instructions among large groups of people. Motivation theory and research, too, provides a rich literature on potential patient-centered factors that might moderate the need for or the effects of interventions. The field of computer-mediated communication (CMC) investigates the effects of communications technologies on communication quality and human relationships.

**Figure 1:**
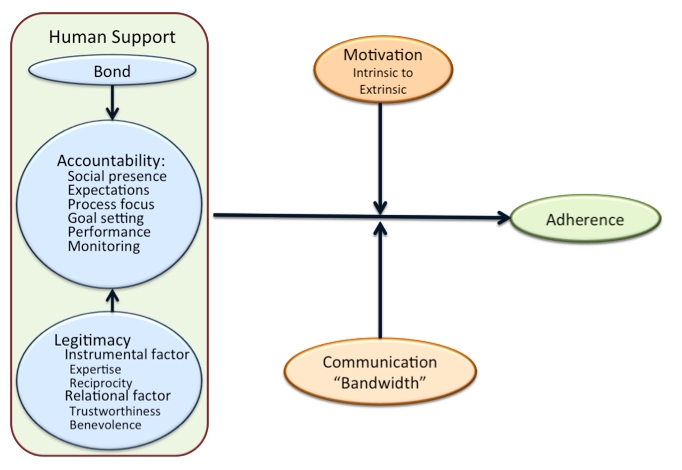
Model of Supportive Accountability

Based on these three literatures, we have constructed a hypothesized model for the factors that explains how human coaches can influence adherence to eHealth interventions. This model, which we call Supportive Accountability, is depicted in Figure 1. Below we will describe each of these factors.

## Accountability

Organizational psychology has focused on questions of how to motivate people to engage in specific behaviors. One area that has focused specifically on adherence is the literature on the use and misuse of accountability in encouraging specific goal-directed behaviors. The term *accountability* refers to the implicit or explicit expectation that an individual may be called upon to justify his or her actions or inactions [22]. The literature identifies several factors that are integral to how accountability is cultivated and maintained.

## Social Presence

Accountability requires *social presence*—the presence of another human being. This presence can be in person, by telephone, or by email, and may be either synchronous or asynchronous. Although it is true that automated systems that monitor and encourage adherence, such as email reminders, can improve adherence to eHealth interventions, human support enhances adherence to a significantly greater degree [6,23,24].

## Expectations

Clarity regarding the *expectations* of patients facilitates adherence. Adherence is not possible when expectations are unknown or unclear. In addition, the reasons for the behavior should be clear. The more that people understand and agree with the underlying rationale for the expected behavior, the greater the compliance [22]. Similarly, in supported e-mental health interventions, when there is agreement between coaches and patients, outcomes are likely to be better [25].

The targets of the expectations can vary. Accountability theory indicates two distinct types of expectations: *o* *utcome* accountability and *process* accountability. For example, outcome accountability for a depression treatment might be defined in terms of depression severity, while process accountability might be defined in terms of completion of thought records or number of logins to a website. Research in accountability fairly consistently finds that process accountability increases completion of the target behaviors, while outcome accountability has primarily detrimental effects, including lower adherence and greater distress. The poor results of outcome accountability are attributed to the effect it has in increasing a desire among people to perform better without respect to the tools and control that could be used to accomplish the goal [22,26,27]. Thus, patients would be much more likely to view feedback from a coach as helpful and rewarding when it is based on the process variables—what they actively do on a session-by-session basis—rather than on a more distal outcome that is not directly under their control.

Another important component of expectations is *goal setting*, which is an important component of many behavioral interventions [28]. However, a review of literature from organizational psychology indicates that goal setting in the context of accountability can have iatrogenic effects in at least two ways [29]. First, goal setting can narrow the focus of behavior onto the specific goals and reduce other behaviors that are useful or important. For example, a clear goal of logging in to an eHealth site 3 times a week may help some patients achieve that goal. However, it may also focus the patient on logging in, leading to perfunctory use of the intervention as opposed to more engaged use. Rigid adherence goals may actually reduce helpful behavior change outside of the narrowly targeted goals of adherence. Adherence goals attached to the patient’s larger goals and values may avoid the iatrogenic effects of goal setting. This might link the tools or content of the materials to be reviewed to a larger goal or value that the patient has. Second, if goals are perceived as being set and monitored by the coach, they may be perceived as controlling, producing a boomerang effect in which the goal behavior is reduced. This is not to say goal setting is always detrimental; rather, we raise this to indicate that goal setting can have negative consequences when not managed properly. The role of goal setting and the form it takes in adherence to eHealth interventions remains an area to be explored.

It is important that expectations be set and agreed upon prior to the point of accountability. Cognitive dissonance theory suggests that once people have committed themselves to a decision or a course of action, learning of the need to justify the action will motivate cognitive effort [30]. However, this cognitive effort will be directed toward self-justification rather than to self-reflection. Thus, if someone learns that he or she will be expected to account for an action or inaction at the time one is to be held accountable, accountability will likely prompt “defensive bolstering,” in which they will rationalize their action or inaction. At that point, the opportunity to help the individual engage in self-reflection likely has been lost. This would argue that it is important to be clear about the accountability process prior to its implementation. Additionally, when expectations of individuals’ roles in the intervention have been clearly and explicitly articulated and agreed upon in advance of the point of accountability, they are more likely to engage in preemptive self-examination of contributing factors [31]—that is, to more effectively explore their cognitions and behaviors on tasks likely to be relevant to the presenting problem but not falling directly under the umbrella of adherence-based goals.

## Performance Monitoring

A core requirement of accountability is that performance be monitored. Paradoxically, however, performance monitoring and surveillance can reduce compliance and contribute to demoralization [22]. The effects of performance monitoring are particularly damaging if surveillance is perceived as controlling and is not accompanied by adequate explanation [32,33]. These negative effects can be mitigated if a clear explanation is provided in advance, and if it is framed in a benevolent context. It should be made clear that the aim of performance monitoring is to provide feedback, that failure to meet goals provides opportunity for self-reflection and growth, and that there are no negative consequences.

Some clinical populations, such as those with depression or severe mental illness, are at particular risk for deterioration, suicide, or negative outcomes. Coach monitoring procedures should also entail monitoring for signs of these outcomes for the protection of these patients.

## Legitimacy

People respond more positively to accountability demands from a coach who is perceived as legitimate [34]. *Legitimacy* stems from patient perceptions about the coach, which dictate that the patient will voluntarily accept the influence of the coach even in the absence of other extrinsic inducements such as reward or punishment. Legitimacy arises from both instrumental and relational factors [34,35].

The instrumental factor has two components. First, legitimacy requires that the patient perceive the coach as having the requisite *expertise*. Perceptions of expertise can be displayed in the interaction by demonstrating knowledge and answering questions, as well as outside the relationship through the display of degrees, certifications, or training [36]. Second, evaluation of the legitimacy of the coach, and adherence that flows from the attribution of legitimacy, rest in part on expectation of *reciprocity*. In its broadest sense, legitimacy rests in part on the expectations of resources to be received and expended in the future, as the relationship develops over time. The contract between patient and coach includes a defined patient role (eg, logging in to a website and performing specific activities), as well as a defined coach role (eg, providing time, attention, and assistance with problems).

The relational component of legitimacy centers on *trustworthiness* and *benevolence* [34]. People seek evidence of integrity, caring, and a sense that the coach has the patient’s best interest at heart when determining legitimacy. The instrumental and relational factors must both be present for coach legitimacy to be established, as well as for adherence to flow from it.

Demands for accountability made by individuals perceived as illegitimate not only fail to produce the desired effects, but may also boomerang [22]. If people perceive that the coach wants to control their beliefs or behaviors, the underlying need for autonomy and freedom of choice is threatened. This activates motivational states aimed at recapturing perceived autonomy, which increases the likelihood of noncompliance with instructions [29,37].

Legitimacy must be both created and sustained. Legitimacy can be cultivated even before the first contact. For example, the credibility of the website may contribute to the creation of coach legitimacy through association. Credibility, which is a characteristic of websites that relies on similar constructs of expertise and trustworthiness [38,39], can be conveyed through the website source (eg, a known university vs an unknown company), presentation (eg, a professional look), names of people associated with the site (eg, recognized experts vs unknown individuals), and design characteristics that are attractive and usable. As we will discuss below, patients likely begin the relationship with a positive bias; however, relatively small negative cues may be overinterpreted, which can quickly undermine coach legitimacy [40]. Coach legitimacy, once created, then must be sustained; this may be accomplished by meeting the agreed-upon expectations for coach behaviors (eg, calling and emailing at the appointed times) or by interacting in ways that consistently convey caring and expertise, among other strategies.

## Bond

The conceptualization of legitimacy is similar to Bordin’s seminal model of therapeutic alliance, which emphasizes liking, trust, and respect [41]. However, legitimacy differs from alliance in several ways. First, legitimacy theory relies on the relational and instrumental factors that are tied to the acceptance of influence in order to achieve a desired outcome. In contrast, Bordin’s notion of a healthy alliance does not hinge on the existence of these same factors in establishing legitimacy. The second fundamental difference between legitimacy and therapeutic alliance is that legitimacy models do not necessarily include liking, or bond. This difference may stem in part from the nature of the goals and interpersonal interactions in psychological intervention versus the goals and relationships that are the focus of organizational psychology. Therapeutic bond is an important predictor of outcome in distance treatments (eg, internet or telephone-administered treatments), particularly when those treatments focus on providing skills training [42]. Accordingly, the emotional attachment captured by the notion of bond likely enhances the effects of accountability.

## Summary of Human Support Constructs

This model of Supportive Accountability suggests that the potential success of accountability is fragile and must be managed carefully. Our model predicts that adherence to prescribed behaviors will be enhanced when (1) coaches are seen as trustworthy and benevolent, (2) coaches are perceived as having the necessary expertise, (3) coaches frame the relationship as one containing reciprocity, in which the patient can expect to receive definable benefits from the coach, (4) coaches involve the patient in the definition of goals and expectations, (5) outcomes for which the patient is accountable are clear, but are also tied to larger life goals and values, (6) expectations are focused on processes rather than outcomes, (7) negative effects of goal setting, such as limiting desired behaviors or inducing perfunctory adherence, are monitored and minimized, (8) coaches are specific about accountability processes at the beginning of treatment, and (9) performance monitoring is introduced with adequate justification and patient agreement, is framed in terms of benefit to the patient, and is devoid of implied threats of negative consequences.

## Motivation

A growing body of data from RCTs shows that adherence to eHealth interventions varies widely [3]. Some percentage of a population is successful and adherent to standalone interventions. Some percentage of a population likely is nonadherent regardless of the quality and amount of support. And the majority likely fall somewhere in between.

Motivation can be defined as that which gives behavior its direction or goals, and determines the strength or energy behind that behavior. Thus, motivation to use an eHealth intervention might be defined by patient and environmental factors that influence whether a person initiates or engages with a website mobile device (goal) and, if so, how frequently he or she uses it (intensity). Self-determination theory is a well-researched theory of motivation that posits that people have innate tendencies for growth and improvement [43]. While self-determination theory focuses on self-determined, intrinsic motivation, it also incorporates extrinsic factors that explain how intrinsic motivation can be modified by external causes. Because self-determination theory sees the determinants of motivated behavior as lying on a gradient from intrinsic to extrinsic, this theory fits well in explaining the variability in adherence seen in supported and standalone eHealth interventions.

*Intrinsic motivation* refers to autonomous, self-determined action that arises out of an innate propensity to seek out and master challenges, to engage and work toward goals, and to be the agent of one’s own life [43]. It arises spontaneously from the individual’s psychological needs, personal curiosity, and innate striving for growth.

Patients in face-to-face psychotherapy generally tend to have better outcomes when they exhibit greater intrinsic motivation [44]. However, people with high intrinsic motivation may be able to use information provided without a therapist. Self-guided treatments have been examined for many behavioral and psychological targets, such as depression, anxiety, diet, physical exercise, smoking cessation, and substance abuse. These interventions have been provided using bibliotherapy as well as unsupported eHealth interventions. Recent meta-analyses found a significant, albeit small, effect for self-guided treatments compared to control conditions [5,45], and found that about 1 in every 8 or 9 participants in these interventions clearly benefits from it. Only a small minority of patients have sufficient intrinsic motivation to be able to successfully implement and sustain the use of self-guided material. For most patients, some extrinsic motivation is required.

*Extrinsic motivation* refers to the motivation that arises from sources external to the individual [43]. Self-determination theory posits that when individuals are more autonomously engaged in a treatment, they are more likely to integrate learning and behavior change, and are more likely to improve. To the extent that people experience their motivation as being a function of external factors, their need for autonomy is threatened and they are more likely to experience conflict and division, and therefore are less likely to comply with the behavioral prescription [37] Furthermore, any change that arises from extrinsic motivation will be unstable and less likely to be maintained once the extrinsic motivators are removed. To cultivate more persistent change, extrinsic motivation must be substituted over time by intrinsic motivation.

The degree to which external motivational factors can be internalized varies along a gradient of autonomy [46]. *External regulation* refers to motivation that is fully extrinsic, such as an external authority that mandates a behavior or compliance with rules, enforced through consequences. *Introjection* refers to esteem-based motivations derived either from seeking social approval or from threats to one’s social-self, such as “shoulds,” guilt, and shame. *Identification* is more on the intrinsic end of the scale, and involves acting in accordance with one’s own values and goals. Pure *intrinsic motivation* is evidenced by activities that are done out of open curiosity, out of interest, or for pure enjoyment. When intrinsic motivation is lacking, motivation to engage in treatment-related behaviors must be enhanced or created, and then it must be sustained. The coach should seek to move the patient along the gradient toward more intrinsic motivation. The more a patient internalizes responsibility for the treatment process, the greater the likelihood of long-term success.

A large body of literature has examined two classes of external motivators: (1) tangible rewards or incentives, and (2) verbal rewards or positive feedback. Tangible rewards such as money may improve outcomes for tasks that are unpleasant, dull, or boring, particularly if the reinforcement is administered variably [47]. However, for tasks that are interesting to the individual, tangible rewards can undermine intrinsic motivation and reduce the maintenance of any behavior change linked to reinforcement for performance of, completion of, or engagement in tasks [48]. One of the reasons that tangible rewards have a negative effect on interesting tasks is that the effect of the reward is mediated by cognitive attributions. That is, the reward itself does not affect behavior; it is the interpretation of the reward that has an effect. Tangible rewards tend to be viewed as indicators that the individual lacks intrinsic motivation or—worse—as controlling and threatening to an individual’s autonomy. Thus, for tasks that the patient may have some interest in completing, tangible rewards may undermine performance.

Verbal rewards, on the other hand, have consistently been found to enhance intrinsic motivation in adults (but not in children) under a broad range of contexts [48]. This is particularly true if positive feedback is provided in a way that affirms competence and is not experienced as controlling. The effectiveness of verbal rewards may stem from their often variable form and timing, thus being a form of variable reinforcement. However, if verbal rewards are offered in a controlling manner, they can undermine intrinsic motivation much as tangible rewards do [49].

## Summary of Motivational Constructs

Although intrinsically motivated adherence to the immediate goals of an eHealth intervention may be difficult to obtain fully for most people, a coach should aspire to help patients identify with the goals of the intervention. The literature on self-determination theory has several direct implications for coach-supported eHealth interventions [48]: (1) a fundamental requirement for any level of intrinsic motivation is that the eHealth intervention should address a problem that the patient has also identified, and should offer some method of resolving that problem, (2) the eHealth intervention and tasks should be constructed to be engaging and interesting, (3) to the degree that the patient does not find the e-intervention tasks interesting, the coach should seek to increase the patient’s level of interest—for example, by increasing the salience of tasks to the patient, helping the patient see the utility and applicability of online tasks to their lives, and enhancing a sense of personal challenge in the completion of tasks, (4) tangible rewards should be avoided, particularly if the targeted activity is interesting to the patient, (5) the patient should be verbally rewarded by acknowledging good performance and good effort, without seeking to control behavior, (6) overt or covert pressure should be avoided, (7) choice regarding how to complete tasks should be provided, and (8) the amount of human support provided by the intervention should be tailored to reflect a patient’s individual orientation on the intrinsic-extrinsic gradient.

Self-determination theory suggests two amendments to accountability theory. First, self-determination theory suggests that intrinsic motivation is more effective than extrinsic motivation in achieving desired behavior, and that the resulting behavior will be more durable. For this reason, motivation in Figure 1 is depicted as a moderator. Patients with high levels of intrinsic motivation may have no need of coaching support at all. For the remaining patients, the processes of accountability are more likely to be successful if they are internalized by the patient. This suggests that adherence will be highest if adherence behaviors are self-monitored, with coaches relegated to roles supporting the patient’s self-monitoring. In other words, when presented with nonadherence, coaches assist patients by reminding them of their personal objectives, promoting self-reflection and problem solving, and providing the socially facilitative relationship through which these processes can unfold.

The second implication of self-determination theory is that application of support and accountability procedures has a threshold, after which additional support either will not add to improvement or may even reduce adherence. Self-determination theory predicts that, while a patient is struggling with adherence, he or she may perceive social facilitation through accountability as helpful. But once adherence and engagement are achieved, the relational context shifts and the patient would be expected to interpret continued support either as controlling or as an indicator that the coach is concerned about the patient’s ability or competence. Thus, patients receiving coaching support after reliably achieving adherence may obtain no further benefit from added support or, worse yet, might show diminished adherence and lower maintenance adherence of therapeutic gains after the removal of the coaching support.

## Computer-Mediated Communication

More than 2 decades of research into CMC has examined the influence of communications media on interpersonal relationships. As with much of the literature discussed in this paper, the CMC literature is based on controlled laboratory research outside the clinical arena. One of the earliest and most straightforward approaches, sometimes referred to as *cues filtered out* [50], suggests that bandwidth is the principle feature affecting communication and the experience of social presence in the communication partner. Bandwidth refers to the number of communication cues a medium can convey (eg, verbal content, visual cues, prosody). The assumption was that greater bandwidth would lead to greater ability to complete tasks, better interpersonal relations, and greater social presence. Thus, face-to-face communication, with its full complement of verbal, nonverbal, and contextual cues, could be assumed to provide the richest source of information. The telephone removes visual cues but retains nonverbal information found in prosody. Instant messaging is primarily content, and would be expected to strip away nonverbal information. Texting and email would eliminate the social presence provided by synchronous communication. Thus, as communication media degrade the quality of the interaction, factors such as bond, legitimacy, and the ability to provide supportive accountability would be expected to deteriorate.

However elegant this formulation is, the CMC literature has since suggested it to be overly simplistic. With time, people are able to develop communications that are effective, and emotionally and relationally rich, even in comparatively lean communications media. Indeed, American teenagers now spend almost as much time in text-based communication (text/chat) as they do in face-to-face and telephone communication, suggesting that these media can provide valued forms of communication [51].

One reason that lean media are effective is that people tend to form stronger impressions based on more limited, sometimes stereotyped social and interpersonal cues. Some of these cues may even be independent of the interaction, such as knowledge about the other person’s gender, status, or other characteristics available to the person [52]. Early in interactions using lean media, people usually make more positive, idealized attributions of their communication partners. This positive effect is heightened when there is an expectation of future contact [40]. When making attributions about communication partners, people using lean media make attributions based on less detailed information, but their attributions tend to be stronger and more intense than those of people communicating face-to-face [53].

The language that people use in text-based communication may also be different from language used in verbal communication. In general, people tend to be more willing to engage in socioemotional communication in text-based media than in face-to-face communication. For example, CMC users employ more self-disclosure than in face-to-face communications [54]. When using CMC, people are also more willing and more likely to ask personal questions, with those questions involving greater depth; questions asked in face-to-face communication are comparatively impersonal and are marked by more superficiality. Ratings of communication effectiveness are also significantly more positive for CMC than for face-to-face. Thus, while face-to-face communication is richer in the availability of cues, people make much more use of the remaining cues and strategies in leaner communication media.

When people do not have nonverbal cues available, they are quite adaptive in developing new methods of creating impression-bearing, interpersonal cues and strategies. Examples include the use of emoticons, such as “;-)”, and abbreviations, such as LOL (laugh out loud), as methods of conveying interpersonal and emotional information. Although people use these frequently to convey such information, findings suggest that they have little effect on a reader’s interpretation of a message [40]. However, when two people engaged in communication mirror the use of emoticons and abbreviations, they are more likely to experience high levels of mutual trust [55]. People also use time and date stamps on CMCs as indicators of the quality of the relationship. For example, task-oriented emails sent at night tend to be perceived as expressing dominance, while personal messages sent during the day tend to be perceived as expressing affection [40]. Longer delays in returning mail may also be perceived as expressing lack of affection.

Entrainment, the process of linguistic and paralinguistic mirroring in dyadic communication, has generally been shown to be associated with more positive relational qualities [40]. This is likely in part because people are more comfortable when they perceive others as being like them [56]. When language shows high similarity in content, people are likely to show higher affiliation and trust. Even the use of similar tenses is associated with greater trust [55]. This suggests that coaches should try, within reason and within constraints established by the legitimacy principle, to mirror their clients in content and tone. Thus, a communication about future plans is best met with a question about those future plans. If it is met with questions about the past, it may be more likely to threaten trust. However, there are some limits to entrainment. For example, entrainment in expressions of negative emotions is associated with decreased trust.

While interactions via CMC have the potential to be more emotional, they also have the potential to be more carefully crafted. Users of asynchronous or text-based media often exploit the absence of cues to more purposefully craft their self-presentation [40]. People use more time to consider whether messages reflect the information and characteristics that they wish to convey. Users also may time self-revelations to manage and serve relational goals. Indeed, the very absence of multiple, simultaneous cues from a partner and lack of environmental stimuli can heighten attention to the targeted integration of socioemotional and task-oriented content. Thus, while CMC can allow patients to be more expressive, and potentially more disclosing, it also affords patients greater ability to engage in impression management. Because cues can take on greater significance in lean communications media, subtle indications from a coach could potentially have a strong effect in shaping the information and quality of patient communications.

While much of the research has examined ways in which the “hyperpersonal” effects of leaner communication media can positively influence communication, negative effects have also been noted. The lack of cues in leaner media means that communication is more effortful [40] and thus requires more time. When time is restricted, the likelihood of negatively interpreted responses increases. Furthermore, the positive bias that is present when beginning communication over lean media is coupled with the expectation of future interactions. These positive biases tend to vanish when there is no expectation of future interaction.

Perhaps because the positive bias is supported by greater reliance on less detailed information, the potential for information to affect the relationship negatively is also greater in lean media than in face-to-face communication. Negative communications, or communications that are perceived as not exhibiting sufficient trust, benevolence, and bond, may have a greater negative impact in leaner media than in face-to-face communications. But even cues that simply provide extraneous information have the potential to negatively affect relationships in lean media. For example, providing photographs of pairs of individuals engaged in long-term CMC reduces positive affect, compared to pairs of individuals who do not receive photographs of their communication partners [57].

Of course, people outside of controlled communications experiments are typically not constrained to communicate solely through one medium. Some of the findings described above may be exaggerated, since the experience of psychological closeness is likely to be enhanced when there are no alternatives to communicating via a lean medium, and may be reduced using a lean medium when other richer media are available [58]. People may also prefer some media over others for specific purposes. For example, media with less social presence are often preferred for more conflictual situations. In addition, people may use different media in sequences or combinations to accomplish certain goals. For example, email is often used to raise an issue prior to a telephone or face-to-face meeting. Thus, a choice of medium that is suboptimal by itself may make sense as part of a larger strategy.

## Summary and Implications for Coaching

Part of the strength of leaner media appears to be the desire of users to have positive impressions of the person with whom they are communicating, and the ability to selectively manage the information and cues that are conveyed. This is believed to result in a “hyperpersonal feedback” effect, in which an idealizing receiver of a message sends a selectively positive message, which triggers a selectively positive message in return. Users of leaner media easily and naturally tend to behave in ways that meet their partner’s exaggerated interpersonal expectations. This positive bias also appears to rely on the interpolation of positive qualities when cues are absent. When those absent cues are filled with actual information, as in the case of photographs, the effect of the positive bias may be diminished. This suggests that coaches should avoid providing extraneous information or cues that are not carefully designed to meet the aims of the intervention.

The CMC literature provides a number of suggestions for shaping coach-patient relationships, particularly via leaner media such as email. First, people base initial judgments on limited cues, and the impact that these cues have in lean media is stronger than in richer media. Careful consideration of cue presentation prior to and in the initial stages of communication is warranted. Second, people tend to enter CMC with a positive bias toward interaction partners. In the absence of cues, people generally make positive assumptions about others. This suggests that in designing coaching interventions, investigators and developers should be judicious in releasing cues about coaches. Third, people are more willing to convey emotional information and disclose uncomfortable information via lean media than they are via richer media. This can be harnessed to facilitate discussion of difficult topics, and coaches should be made aware of this possible benefit of CMC to make interactions with patients more efficient. Fourth, people search for cues in lean media. Timing can become an important cue. Responses should be timely. Some CMC responses outside of normal working hours may be viewed as expressions of caring. Fifth, people feel more comfortable with people who are like them. Mirroring the content, style, and even tense of patient communications should be used to promote positive relational qualities. Sixth, CMC allows more time to craft messages. Patients will likely craft messages to please the coach. This tendency should be considered in coach communications. Seventh, leaner communications media sometimes require more time and effort to achieve goals. Coaches should anticipate investing their resources in light of this phenomenon. Eighth, if multiple media are used, the overall strategy should be considered. For example, if coaches can use both email and the telephone, it may be strategic to permit potentially difficult or embarrassing information to initially be provided via email, offer a sympathetic response email to underscore bond and the coach’s benevolence, and then follow up by telephone, which can provide greater social presence.

## Conclusions

The effectiveness of and adherence to eHealth interventions is enhanced by human support [3,5]. Based on our review of the existing literature from organizational psychology, motivational theory, and CMC, we have developed a framework for understanding and constructing human support components of eHealth interventions. We call this model, displayed graphically in Figure 1, Supportive Accountability. Human factors, such as accountability, bond, and legitimacy, can potentially influence adherence to eHealth interventions. However, we posit that the effect of human factors is moderated by motivational factors, as well as the communications media used. This model is based on basic research, and therefore represents our best guess for what will be effective; however, the components of the model have not been tested in clinical interventions. This model and its components are described so as to be testable, with the aim of developing clearly defined, manualized, evidence-based human support programs. The refinement of such human support models has the potential to enhance effectiveness and adherence to eHealth intervention.

## Abbreviations

CMC: computer-mediated communication
RCT: randomized controlled trials
